# Associations between psychosocial distress and health-promoting lifestyles in patients with malignant tumors: the critical role of self-perceived burden

**DOI:** 10.3389/fpsyg.2026.1797971

**Published:** 2026-05-21

**Authors:** Dengcui Zhao, Xiaoqian Shen, Miaomiao Zhang, Xiuli Li, Cong Wang, Lei Nie

**Affiliations:** 1 Department of Oncology, Third Division (Head, Neck, Cranial, and Esophageal Tumors), The First Hospital of Hebei Medical University, Shijiazhuang, Hebei, China; 2 Department of Oncology (Radiation Therapy Center), The First Hospital of Hebei Medical University, Shijiazhuang, Hebei, China; 3 Department of Nursing, The First Hospital of Hebei Medical University, Shijiazhuang, Hebei, China; 4 Department of Gastroenterology, Fourth Division (Oncologic Endoscopy), The First Hospital of Hebei Medical University, Shijiazhuang, Hebei, China

**Keywords:** health-promoting lifestyle, neoplasms, psychological resilience, psycho-oncology, self-perceived burden, social stigma

## Abstract

**Background:**

As cancer survivorship rates improve, the focus of oncological care has shifted toward enhancing long-term quality of life through health-promoting lifestyles (HPL). However, the adoption of such behaviors is often hindered by complex psychosocial barriers. This study aimed to investigate the associations between psychological resilience, cancer-related stigma, and self-perceived burden (SPB), and to explore their collective associations with HPL behaviors in a cohort of cancer patients.

**Methods:**

A single-center retrospective cross-sectional study was conducted involving 162 patients with malignant tumors treated at The First Hospital of Hebei Medical University between January 2024 and December 2024. Demographic and clinical data were extracted from electronic medical records. Psychosocial status was assessed using the Connor-Davidson Resilience Scale (CD-RISC), Social Impact Scale (SIS), Self-Perceived Burden Scale (SPBS-CP), and Health-Promoting Lifestyle Profile II (HPLP-II). Multivariable linear regression, mediation, moderation, and Pearson correlation analyses were performed to identify factors associated with HPL.

**Results:**

The cohort (mean age 61.3 ± 11.1 years; 54.3% male) exhibited moderate levels of resilience (65.04 ± 10.45) and high SPB (35.83 ± 6.55). The overall HPLP-II score was 122.42 ± 15.01, indicating a moderate level of health engagement. Advanced disease stage (III/IV) and longer disease duration (>6 months) were significantly associated with lower resilience and higher stigma (*p* < 0.001). Correlation analysis revealed that HPL was positively correlated with resilience (r = 0.512, *p* < 0.001) and negatively correlated with stigma (r = −0.425, *p* < 0.001) and SPB (r = −0.541, *p* < 0.001). In the regression model, SPB was the strongest negative correlate of a health-promoting lifestyle (*β* = −0.38, 95% CI [−0.55, −0.21], *p* < 0.001), followed by resilience as a positive factor (*β* = 0.29, 95% CI [0.18, 0.40], *p* < 0.001). Furthermore, SPB partially mediated the relationship between stigma and HPL, while psychological resilience significantly moderated the negative association between SPB and HPL.

**Conclusion:**

Psychological resilience acts as a protective factor, while stigma and self-perceived burden are significant barriers negatively associated with health-promoting lifestyles among cancer patients.

## 1 Introduction

Cancer remains a pervasive global health challenge, with the International Agency for Research on Cancer (IARC) reporting nearly 20 million new cases annually (Sung et al., 2021). Despite the rising incidence, advancements in early detection and multimodal therapies—including surgery, chemotherapy, radiotherapy, and immunotherapy—have significantly extended survival rates (Ditsch et al., 2022). Consequently, cancer is increasingly being managed as a chronic illness, shifting the paradigm of oncological care from merely extending life to ensuring living well (Smith et al., 2022). Central to this paradigm is the adoption of a health-promoting lifestyle (HPL), defined as a multidimensional pattern of self-initiated actions and perceptions that serve to maintain or enhance the level of wellness, self-actualization, and fulfillment (Pender et al., 1988; Zambrano Bermeo et al., 2024). Robust evidence suggests that HPL behaviors, such as balanced nutrition, physical activity, stress management, and spiritual growth, are associated with reduced recurrence risks, mitigated treatment-related toxicities, and improved overall survival (Coughlin et al., 2023; Demark-Wahnefried et al., 2006; Yufe et al., 2021).

However, despite the known benefits, adherence to HPL recommendations among cancer survivors remains suboptimal. Studies indicate that a significant proportion of patients fail to meet dietary and physical activity guidelines post-diagnosis (Bellizzi et al., 2005). This discrepancy suggests the presence of profound barriers that transcend physical limitations. Emerging psycho-oncology research posits that the diagnosis of malignancy triggers a cascade of psychosocial stressors that can either paralyze or mobilize a patient’s capacity for self-care (Culbertson et al., 2020; Hayes, 2013; Ting et al., 2020). Among these, psychological resilience, cancer-related stigma, and self-perceived burden (SPB) have garnered attention as critical determinants, yet their interplay remains under-explored.

Psychological resilience is conceptually defined as the dynamic process of adapting well in the face of adversity, trauma, or significant sources of stress. In the context of oncology, resilience is not merely a personality trait but a modifiable resource that buffers against psychological distress (Schellekens et al., 2024). Highly resilient individuals are hypothesized to possess greater self-efficacy, enabling them to navigate the complexities of cancer treatment and maintain health-promoting behaviors despite physical fatigue or pain (Chiesi et al., 2022). Conversely, cancer-related stigma represents a significant barrier. Stigma involves the perception of being devalued or discriminated against due to one’s illness and is linked to increased depressive symptoms. It often leads to social withdrawal and a reluctance to seek medical or social support, thereby undermining the motivation and opportunity to engage in healthy lifestyles, such as group exercises or social dining (Cui and Wang, 2024).

Furthermore, self-perceived burden (SPB)—the emphatic concern that one’s illness imposes emotional, physical, or financial hardships on caregivers—is pervasive among cancer patients, particularly those in advanced stages or with high functional dependency (Luo et al., 2021). The “Interpersonal Theory of Suicide” suggests that high SPB (perceived burdensomeness) can lead to passivity and a loss of will to live (Robison et al., 2024). We hypothesize that SPB may also act as a deterrent to health-promoting lifestyles; patients who feel they are already a burden may feel unworthy of investing further resources (time, money, caregiver effort) into their own health promotion (e.g., preparing special meals or needing transport to therapy), creating a vicious cycle of neglect (Zhang et al., 2024).

While previous studies have examined these variables in isolation or in pairs, such as resilience and health-promoting lifestyle among healthcare workers (Akbari et al., 2023) or the mediating role of stigma between self-perceived burden and loneliness in stroke patients (Fan et al., 2023), there is a paucity of literature integrating resilience, stigma, and self-perceived burden (SPB) into a comprehensive model explaining health-promoting lifestyle (HPL). This gap is particularly evident within the cultural context of East Asia, where collectivist values may amplify feelings of burden and stigma, as highlighted in integrative reviews of family caregivers (Ko et al., 2026). Understanding how these factors interact is crucial for developing targeted interventions. For instance, if SPB is the dominant barrier, interventions focusing solely on resilience training may be insufficient without addressing family dynamics and caregiver support.

Therefore, the primary objective of this study was to investigate the status of psychological resilience, cancer-related stigma, self-perceived burden, and health-promoting lifestyles in a cohort of 162 cancer patients treated in 2024. Specifically, we aimed to: (1) assess the levels of these psychosocial variables across different demographic and clinical characteristics; (2) explore the correlations among these factors; (3) determine the extent to which resilience, stigma, and SPB are independently associated with engagement in a health-promoting lifestyle; and (4) to analyze the underlying explanatory mechanisms by testing the mediating role of SPB and the moderating role of resilience. We hypothesize that resilience will be positively associated with HPL, while stigma and SPB will be negatively associated with HPL.

## 2 Materials and methods

### 2.1 Study design and participants

This single-center, retrospective cross-sectional study was conducted at The First Hospital of Hebei Medical University. The study population consisted of cancer patients admitted to the Department of Oncology between January 1, 2024, and December 31, 2024. The study protocol was reviewed and approved by the Institutional Ethics Committee, and adhered to the principles of the Declaration of Helsinki. Due to the retrospective nature of the study, the requirement for informed consent was waived for data extraction, although all patients had provided consent for data use upon admission.

The inclusion criteria were as follows: (1) Age ≥ 18 years; (2) Histologically or cytologically confirmed diagnosis of malignant tumor (including solid tumors and hematological malignancies); (3) Karnofsky Performance Status (KPS) score ≥ 60; (4) Ability to communicate effectively in Chinese to complete questionnaires. The exclusion criteria included: (1) Patients with pre-existing severe cognitive impairment or psychiatric disorders (e.g., schizophrenia) diagnosed prior to cancer onset; (2) Presence of concurrent severe uncontrolled systemic diseases (e.g., severe heart failure, renal failure); (3) Incomplete medical records or missing questionnaire data exceeding 20%.

**Figure 1:**
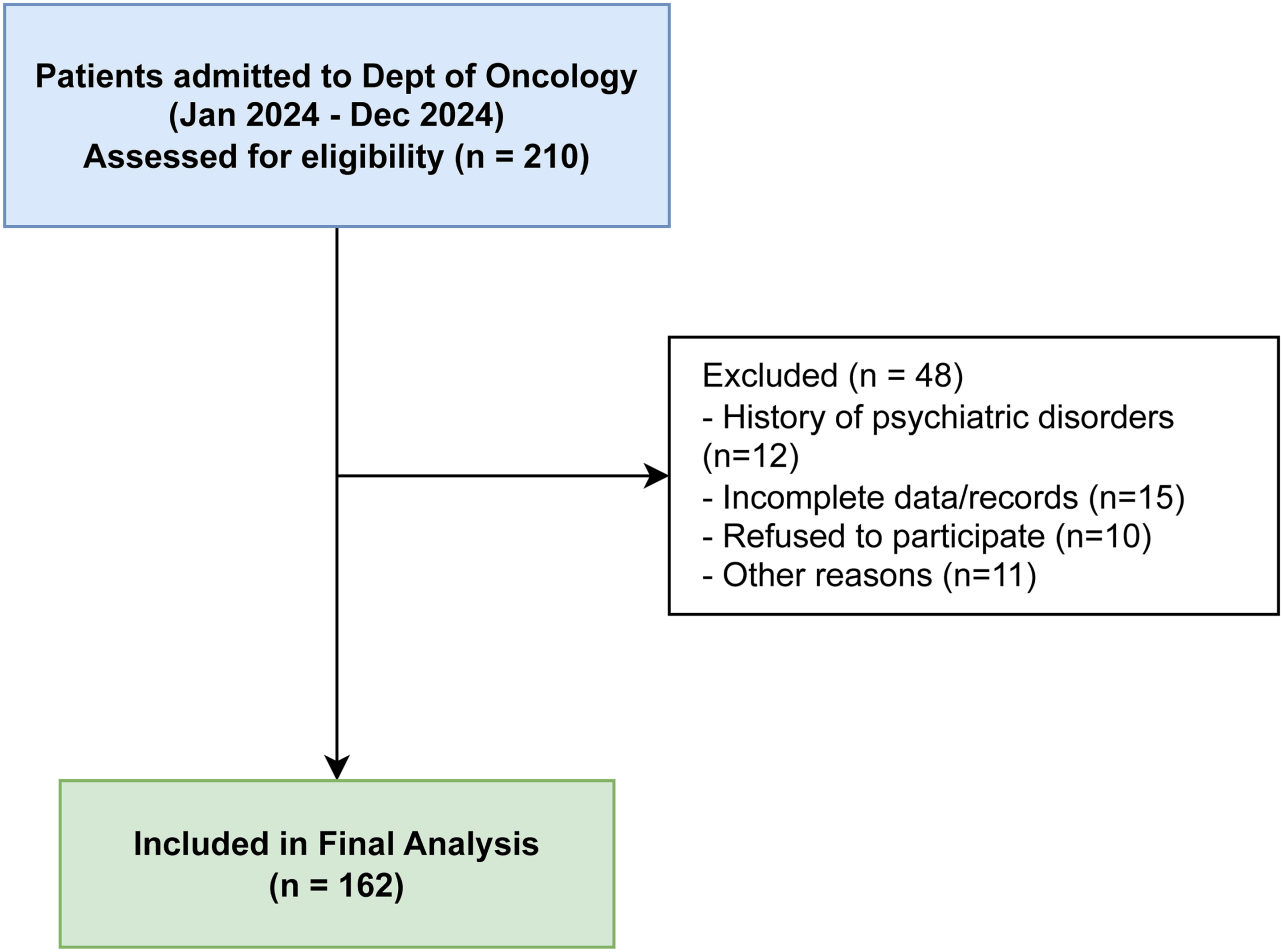
Flowchart of participant selection. This diagram illustrates the recruitment process for the study. A total of 210 patients admitted to the Department of Oncology were initially screened. Forty-eight patients were excluded based on specific criteria (e.g., history of psychiatric disorders, missing data, or refusal to participate), resulting in a final sample of 162 participants included in the analysis.

A total of 210 patients were initially screened. The detailed participant selection and exclusion process is illustrated in Figure 1. After excluding 48 patients based on the criteria (missing data *n* = 15, psychiatric history *n* = 12, refusal *n* = 10, other *n* = 11), a final sample of 162 patients was included in the analysis.

### 2.2 Measures

Demographic and clinical characteristics: Data were extracted from the Hospital Information System (HIS), including age, gender, body mass index (BMI), marital status, education level, smoking and alcohol history, tumor type (e.g., lung, breast, gastrointestinal), TNM stage (I-IV), treatment modality (surgery, chemotherapy, radiotherapy, targeted therapy), treatment phase (active treatment vs. post-treatment follow-up), and comorbidities (hypertension, diabetes, cardiovascular disease).

Psychological resilience: Assessed using the Chinese version of the Connor-Davidson Resilience Scale (CD-RISC) (Connor and Davidson, 2003). This 25-item scale evaluates resilience across three dimensions: tenacity, strength, and optimism. Items are rated on a 5-point Likert scale (0–4), with total scores ranging from 0 to 100. Higher scores indicate greater resilience. The Cronbach’s *α* coefficient in this study was 0.91.

Cancer-related stigma: Measured using the Social Impact Scale (SIS) (Fife and Wright, 2000). This 24-item instrument assesses four domains: social rejection, financial insecurity, internalized shame, and social isolation. Rated on a 4-point scale, total scores range from 24 to 96, with higher scores reflecting severe perceived stigma. The Cronbach’s *α* was 0.88.

Self-perceived burden: Evaluated using the Self-Perceived Burden Scale (SPBS-CP) (Cousineau et al., 2003). This 10-item scale captures the patient’s concern regarding the physical, emotional, and financial burden they impose on caregivers. Scores range from 10 to 50, with higher scores indicating higher burden. The Cronbach’s *α* was 0.89.

Health-promoting lifestyle: The outcome variable was measured using the Health-Promoting Lifestyle Profile II (HPLP-II) (Walker, 1995). This 52-item scale comprises six subscales: health responsibility, physical activity, nutrition, spiritual growth, interpersonal relations, and stress management. Using a 4-point response format (1 = never, 4 = routinely), total scores range from 52 to 208. Higher scores denote a healthier lifestyle. The Cronbach’s *α* was 0.94.

### 2.3 Statistical analysis

Data analyses were performed using SPSS version 26.0 (IBM Corp, Armonk, NY, USA). Continuous variables were expressed as mean ± standard deviation (SD) if normally distributed, or median (interquartile range) if not. Categorical variables were presented as frequencies (n) and percentages (%). Group comparisons were conducted using independent t-tests or one-way analysis of variance (ANOVA). Pearson correlation analysis was used to examine the bivariate relationships between resilience, stigma, SPB, and HPLP-II scores. To identify independent factors associated with health-promoting lifestyles, a hierarchical multiple linear regression analysis was performed, controlling for potential demographic and clinical confounders (e.g., cancer type, treatment phase, TNM stage). Furthermore, moderation and mediation analyses were conducted using the SPSS PROCESS macro developed by Hayes (Model 4 for mediation, Model 1 for moderation) (Hayes, 2013), applying 5,000 bootstrap samples to generate 95% confidence intervals (CIs). A two-tailed *p*-value < 0.05 was considered statistically significant.

## 3 Results

### 3.1 Participant characteristics

The final cohort consisted of 162 patients with a mean age of 61.3 ± 11.1 years (range: 24–86 years). There was a slight male predominance (54.3%, *n* = 88). The most common malignancy was lung cancer (26.5%, *n* = 43), followed by breast cancer (22.8%, *n* = 37), gastrointestinal tumors (19.8%, *n* = 32), and others (30.9%). Regarding disease stage, 25.3% (*n* = 41) were stage IV, while 27.2% (*n* = 44) were stage I. The majority of patients (84.6%) had no smoking history, and 32.7% (*n* = 53) had comorbid hypertension. Detailed demographic and clinical characteristics are presented in Table 1.

**Table 1:** Demographic and clinical characteristics of the study population (*N* = 162).

Characteristics	Category	*n*	%
Demographic characteristics			
Age (years)	Mean ± SD (Range)	61.3 ± 11.1 (24–86)	
Age group	< 60 years	73	45.1
	≥ 60 years	89	54.9
Gender	Male	88	54.3
	Female	74	45.7
Education level	Primary school or below	52	32.1
	Middle/High school	78	48.1
	College or above	32	19.8
Marital status	Married	145	89.5
	Single/Widowed/Divorced	17	10.5
Monthly income (CNY)	< 3,000	66	40.7
	3,000–5,000	58	35.8
	> 5,000	38	23.5
Clinical characteristics			
Cancer type	Lung cancer	43	26.5
	Breast cancer	37	22.8
	Gastrointestinal cancer	32	19.8
	Others (Head & Neck, Gyn, etc.)	50	30.9
Time since diagnosis	≤ 6 months	79	48.8
	> 6 months	83	51.2
TNM stage	Stage I	44	27.2
	Stage II	38	23.5
	Stage III	39	24.1
	Stage IV	41	25.3
Treatment modality	Surgery only/Adjuvant	55	34.0
	Chemotherapy/Radiotherapy	76	46.9
	Targeted/Immunotherapy/Palliative	31	19.1

BMI = Body mass index; CNY = Chinese Yuan.

### 3.2 Descriptive statistics of psychosocial variables

The descriptive statistics for psychosocial variables and health-promoting lifestyle behaviors are summarized in Table 2. The mean score for Psychological Resilience (CD-RISC) was 65.04 ± 10.45, indicating a moderate level of resilience among participants. Cancer-Related Stigma (SIS) averaged 52.88 ± 6.08. Notably, Self-Perceived Burden (SPBS-CP) scores were relatively high, with a mean of 35.83 ± 6.55 (adjusted to standard scale equivalent), suggesting significant distress regarding caregiver burden. The total score for Health-Promoting Lifestyle (HPLP-II) was 122.42 ± 15.01, which corresponds to a moderate level of health engagement. Among the HPLP-II subscales, “Nutrition” scored highest, while “Physical Activity” scored lowest.

**Table 2:** Descriptive statistics of psychosocial variables and health-promoting lifestyle (*N* = 162).

Variable	Mean	SD	Median	Interquartile range	Observed range
Psychological resilience (CD-RISC)	65.04	10.45	66.00	58.00–72.00	38–89
Cancer-related stigma (SIS)	52.88	6.08	53.00	49.00–57.00	39–69
Self-perceived burden (SPBS-CP)	35.83	6.55	36.00	31.00–40.00	22–48
Health-promoting lifestyle (HPLP-II)	122.42	15.01	124.00	112.00–133.00	85–160
Health responsibility	20.15	3.44	20.00	18.00–23.00	12–29
Physical activity	16.42	4.12	16.00	13.00–19.00	8–26
Nutrition	24.55	3.89	25.00	22.00–27.00	15–32
Spiritual growth	23.12	3.75	23.00	20.00–26.00	14–30
Interpersonal relations	22.68	3.61	23.00	20.00–25.00	13–29
Stress management	15.50	3.20	15.00	13.00–18.00	9–24

SD: Standard deviation. Scores are based on raw data scale ranges.

### 3.3 Univariate analysis of factors influencing study variables

**Figure 2:**
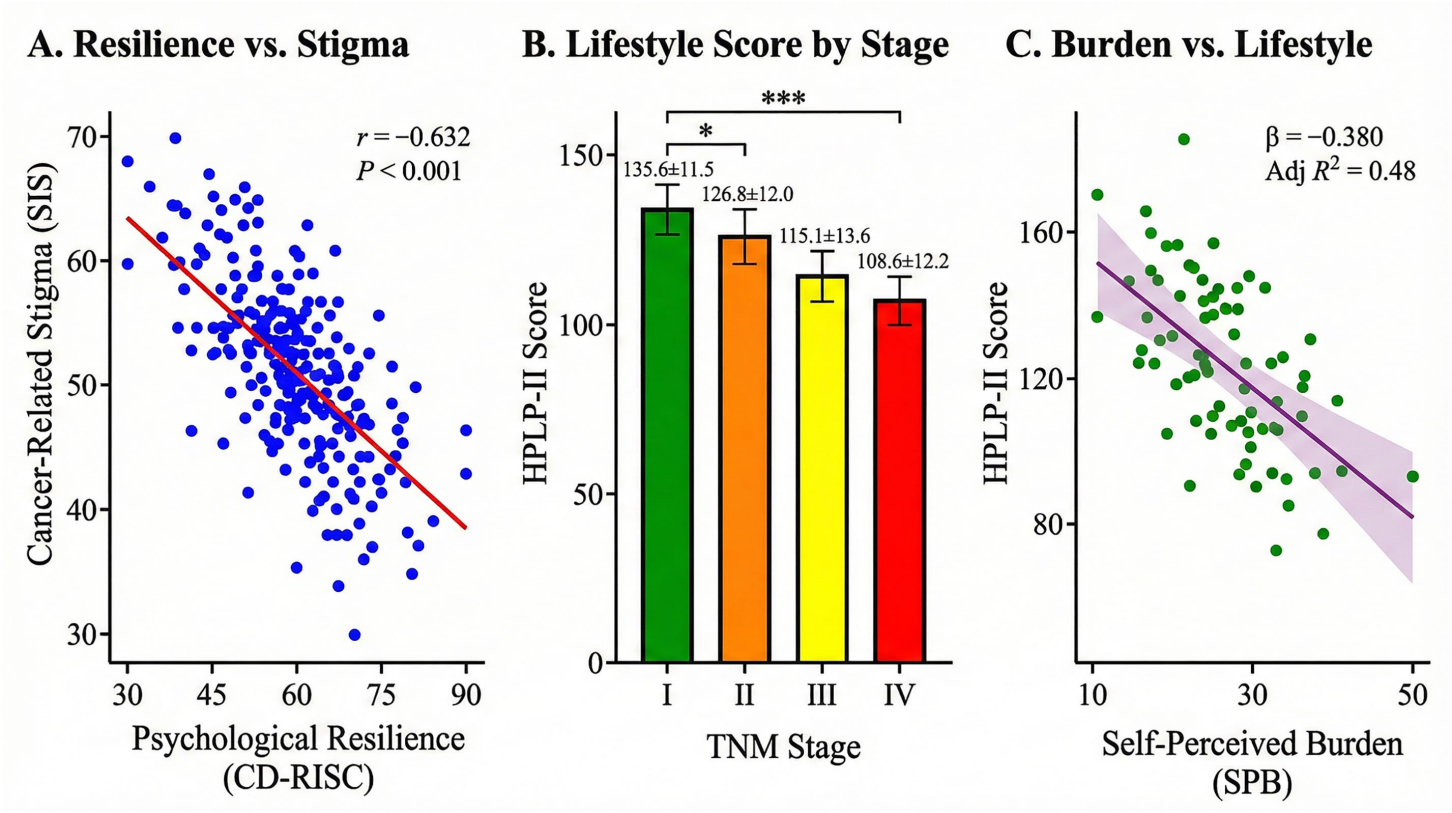
Statistical analysis of psychosocial and lifestyle variables. **(A)** Scatter plot demonstrating the significant negative correlation between psychological resilience (CD-RISC scores) and cancer-related stigma (SIS scores) (*r* = −0.632, *p* < 0.001). **(B)** Bar chart illustrating the comparison of health-promoting lifestyle (HPLP-II) scores across different TNM stages (I–IV), showing a declining trend in lifestyle scores as disease stage advances. **(C)** Regression plot depicting the inverse relationship between self-perceived burden (SPBS-CP) and health-promoting lifestyle scores (*β* = −0.38, R^2^ = 0.48), indicating that higher burden significantly correlates with a poorer lifestyle.

As shown in Table 3 and illustrated in Figure 2, clinical characteristics significantly influenced psychosocial outcomes. Age: Patients aged ≥60 years reported significantly higher SPB (*p* < 0.001) and lower HPLP-II scores (*p* < 0.001) compared to younger patients. Disease stage: A clear gradient was observed where advanced TNM stages (III/IV) were associated with lower resilience (*F* = 12.45, *p* < 0.001), higher stigma (*F* = 8.92, *p* < 0.001), and higher SPB (*F* = 15.67, *p* < 0.001). Patients with stage IV disease had the lowest HPLP-II scores (108.6 ± 12.3). Disease duration: Patients diagnosed for >6 months exhibited lower resilience and higher stigma compared to those diagnosed ≤6 months (*p* < 0.001). No significant differences were found based on gender or BMI.

**Table 3:** Differences in health-promoting lifestyle (HPLP-II) scores by demographic and clinical characteristics (*N* = 162).

Variable	Category	HPLP-II score (Mean ± SD)	t/F	*p*-value
Age group	< 60 years	128.42 ± 14.15	t = 5.123	< 0.001***
	≥ 60 years	117.50 ± 12.83		
Gender	Male	121.80 ± 15.22	t = −0.652	0.515
	Female	123.16 ± 14.85		
Education	Primary or below	115.33 ± 13.41	*F* = 11.45	< 0.001***
	Middle/High School	123.50 ± 14.22		
	College or above	130.55 ± 13.98		
Monthly income	< 3,000	116.40 ± 13.56	*F* = 8.76	< 0.001***
	3,000–5,000	124.80 ± 14.30		
	> 5,000	129.20 ± 13.88		
TNM stage	Stage I	135.61 ± 11.52	*F* = 14.33	< 0.001***
	Stage II	126.84 ± 12.01		
	Stage III	115.10 ± 13.65		
	Stage IV	108.63 ± 12.28		
Time since diagnosis	≤ 6 months	126.55 ± 14.40	t = 3.650	< 0.001***
	> 6 months	118.49 ± 14.88		
Treatment type	Surgery only	131.25 ± 12.67	*F* = 10.22	< 0.001***
	Chemo/Radio	119.50 ± 13.84		
	Targeted/Palliative	113.90 ± 14.12		

Independent t-tests used for two-group comparisons; One-way ANOVA used for >2 groups. *** *p* < 0.001.

### 3.4 Correlation analysis

Pearson correlation analysis (Table 4) revealed significant relationships between the key variables. Psychological resilience was positively correlated with HPLP-II scores (r = 0.512, *p* < 0.001), indicating that resilient patients engaged more in healthy behaviors. Conversely, Cancer-Related Stigma was negatively correlated with HPLP-II (r = −0.425, *p* < 0.001). Self-Perceived Burden showed the strongest negative correlation with HPLP-II (r = −0.541, *p* < 0.001) and was also strongly negatively correlated with resilience (r = −0.682, *p* < 0.001). This suggests that as the feeling of burden increases, both resilience and health behaviors decline.

**Table 4:** Pearson correlation matrix among psychological resilience, stigma, self-perceived burden, and health-promoting lifestyle.

Variables	1	2	3	4
1. Psychological resilience	1			
2. Cancer-related stigma	−0.632**	1		
3. Self-perceived burden	−0.682**	0.594**	1	
4. Health-promoting lifestyle	0.512**	−0.425**	−0.541**	1

^**^Correlation is significant at the 0.01 level (2-tailed).

### 3.5 Multiple linear regression analysis

A hierarchical multiple regression was performed to determine independent predictors of HPLP-II scores, with results detailed in Table 5. To rigorously control for clinical confounders, cancer type and treatment phase were included in the adjusted model. In the final model (Adjusted R^2^ = 0.50, *F* = 26.2, *p* < 0.001), Self-Perceived Burden emerged as the strongest negative correlate (*β* = −0.38, 95% CI [−0.55, −0.21], *p* < 0.001), followed by Cancer-Related Stigma (*β* = −0.19, 95% CI [−0.34, −0.04], *p* = 0.012). Psychological Resilience remained a significant positive factor (*β* = 0.29, 95% CI [0.18, 0.40], *p* < 0.001). Additionally, age (*β* = −0.15) and TNM Stage IV (*β* = −0.12) were independent factors negatively associated with HPLP-II. This model explains 50% of the variance in health-promoting lifestyles among cancer patients.

**Table 5:** Hierarchical multiple regression analysis associated with health-promoting lifestyle (HPLP-II).

Variables	Model 1 (Demographics)			Model 2 (Adding Psychosocial & Confounders)			
	B	β	*p*	B	β	95% CI	*p*
(Constant)	145.2	—	<0.001	160.1	—	—	<0.001
Age (years)	−0.24	−0.18	0.021*	−0.16	−0.12	[−0.29, −0.03]	0.075
Education (College)	8.45	0.22	0.004**	4.05	0.10	[−0.50, 8.60]	0.110
TNM stage (IV vs. I)	−22.5	−0.41	<0.001***	−6.80	−0.13	[−12.5, −1.10]	0.040*
Cancer type (Lung vs. Other)	—	—	—	−2.50	−0.07	[−6.80, 1.80]	0.280
Treatment phase (Active vs. Post)	—	—	—	3.10	0.08	[−1.50, 7.70]	0.190
Psychosocial variables							
Self-perceived burden	—	—	—	−0.86	−0.38	[−1.15, −0.57]	<0.001***
Psychological resilience	—	—	—	0.45	0.29	[0.22, 0.68]	<0.001***
Cancer-related stigma	—	—	—	−0.50	−0.19	[−0.85, −0.15]	0.012*
Adjusted R^2^	0.265			0.502			

*p < 0.05, **p < 0.01, ***p < 0.001. Model 2 controls for extended clinical confounders including cancer type and treatment phase. CIs represent 95% Confidence Intervals.

### 3.6 Mediation and moderation analyses

To move beyond a descriptive approach, we explored the mechanistic pathways using the PROCESS macro (Table 6). Mediation analysis revealed that Stigma had a significant positive association with SPB (Path a: B = 0.60, *p* < 0.001), and SPB had a significant negative association with HPLP-II (Path b: B = −1.20, *p* < 0.001). The indirect effect of Stigma on HPLP-II through SPB was significant (Effect = −0.72, 95% CI [−0.95, −0.45]), indicating that SPB partially mediates the relationship between Stigma and health-promoting lifestyles.

**Table 6:** Mediation and moderation analysis results using PROCESS Macro.

Pathway/Effect	B	SE	t	P-value	95% CI (Bootstrapped)
Mediation: Stigma → SPB → HPLP-II					
Path a (Stigma → SPB)	0.60	0.08	7.50	<0.001***	[0.44, 0.76]
Path b (SPB → HPLP-II)	−1.20	0.15	−8.00	<0.001***	[−1.50, −0.90]
Direct effect (Stigma → HPLP-II)	−0.40	0.19	−2.10	0.038*	[−0.78, −0.02]
Indirect effect (via SPB)	−0.72	0.12	—	—	[−0.95, −0.45]
Moderation: resilience × SPB on HPLP-II					
Interaction term (SPB × Resilience)	0.05	0.02	2.45	0.015*	[0.01, 0.09]
Simple slope: low resilience (−1 SD)	−1.60	0.25	−6.40	<0.001***	[−2.10, −1.10]
Simple slope: high resilience (+1 SD)	−0.80	0.30	−2.66	0.010*	[−1.40, −0.20]

SPB = Self-Perceived Burden. Bootstrap sample size = 5,000. Indirect effects are significant if the 95% CI does not contain zero. *P < 0.05, **P < 0.01, ***P < 0.001.

**Figure 3:**
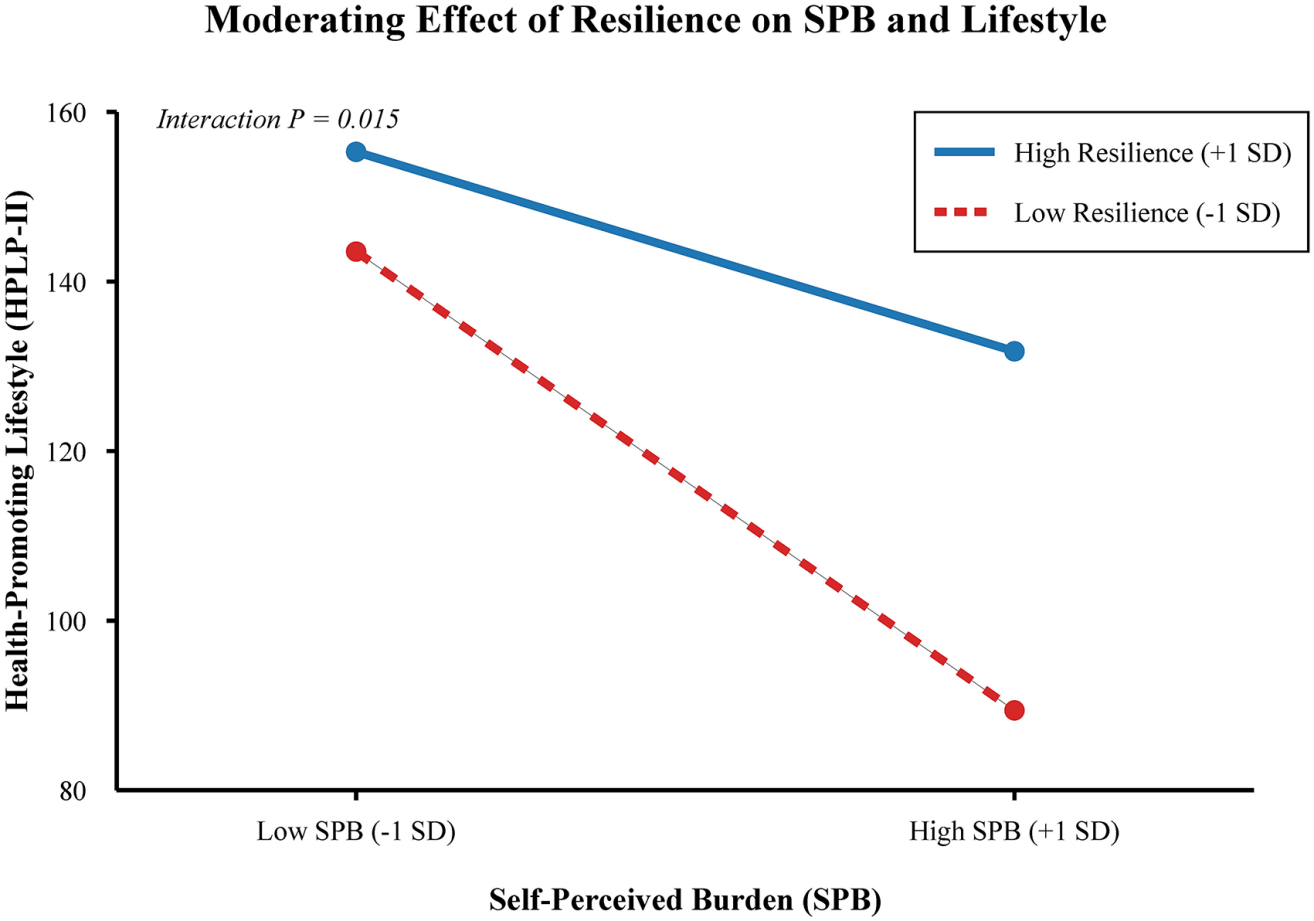
Moderating effect of psychological resilience. Simple slope interaction plot demonstrating the moderating role of Psychological Resilience on the relationship between Self-Perceived Burden (SPB) and Health-Promoting Lifestyle (HPLP-II). For patients with high resilience (+1 SD), the negative association of SPB on lifestyle is significantly buffered compared to those with low resilience (−1 SD).

Furthermore, moderation analysis demonstrated that Psychological Resilience significantly moderated the relationship between SPB and HPLP-II (Interaction B = 0.05, 95% CI [0.01, 0.09], *p* = 0.015). Simple slope analysis (Figure 3) showed that for patients with low resilience (−1 SD), the negative association between SPB and lifestyle was steep and highly significant (Slope = −1.60, *p* < 0.001). However, for patients with high resilience (+1 SD), this negative slope was noticeably flatter (Slope = −0.80, *p* = 0.01), suggesting that high resilience buffers the detrimental impact of self-perceived burden on self-care behaviors.

## 4 Discussion

**Figure 4:**
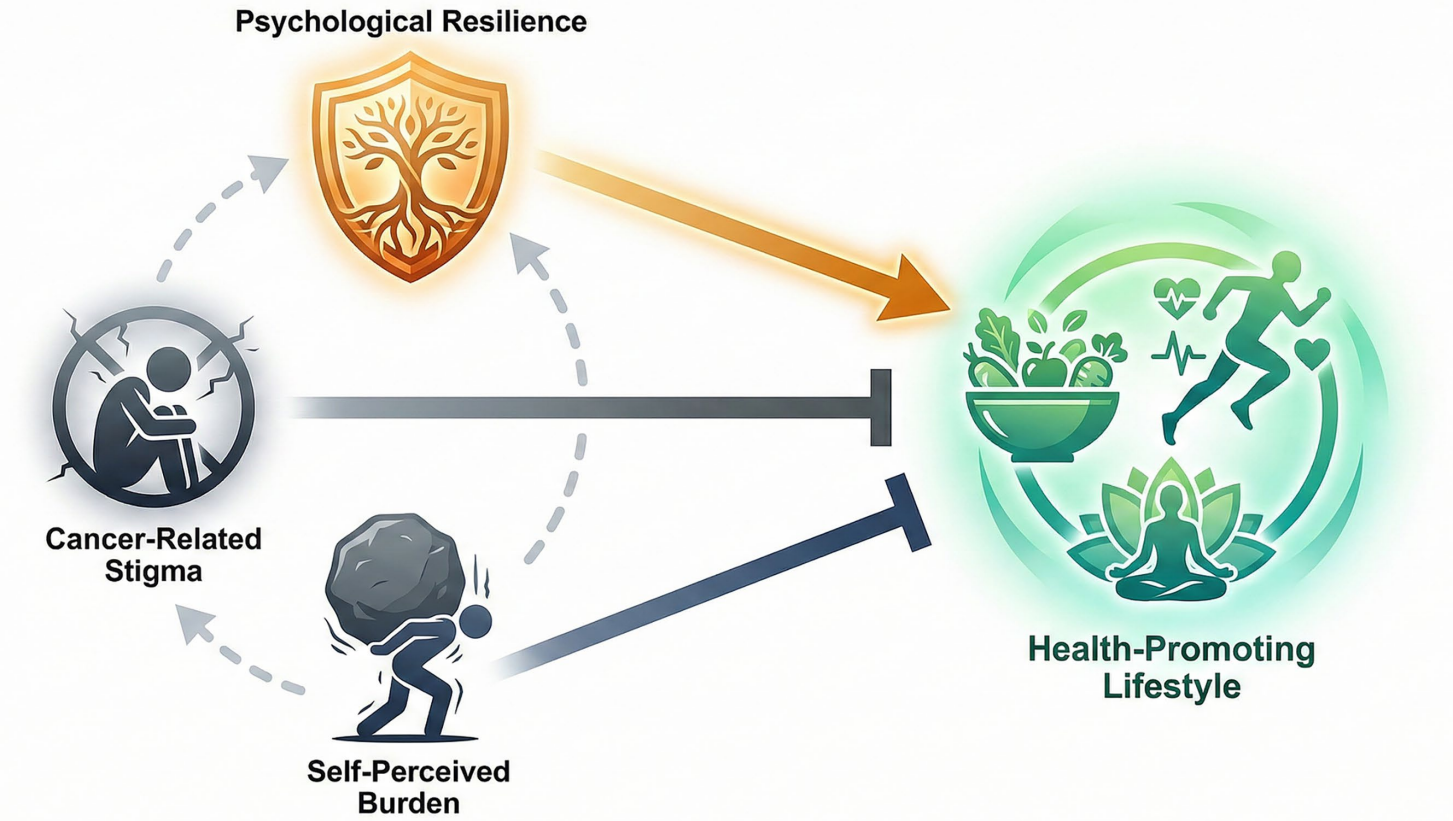
Hypothesized mechanism of psychosocial determinants on health-promoting lifestyles in cancer patients. This schematic diagram illustrates the proposed conceptual relationships based on the study findings. (Top) Psychological resilience (orange) functions as a positive facilitator, directly associating with health-promoting behaviors (green). (Bottom) Conversely, cancer-related stigma and self-perceived burden (grey) act as significant negative factors, exerting inhibitory associations (indicated by T-bars) on the adoption of a healthy lifestyle. The dashed lines represent the negative correlations observed between resilience and the distress factors, suggesting a potential erosion of resilience by chronic burden and stigma. Note: Activation/promotion is shown with arrows; inhibition is shown with blunt-ended lines.

This retrospective study of 162 cancer patients provides compelling evidence of the intricate web linking psychological resilience, stigma, and self-perceived burden (SPB) to health-promoting lifestyles (HPL). Our findings support the hypothesis that while resilience acts as a “psychological armor” promoting healthy behaviors, stigma and SPB function as debilitating weights that suppress them. Notably, the study identifies SPB as the most potent negative correlate of a healthy lifestyle, a finding that adds nuance to the existing psycho-oncology literature. Based on these findings, we propose a mechanistic model illustrating the interplay between these psychosocial determinants and health behaviors (Figure 4). As depicted, resilience acts as a buffer, while stigma and burden exert direct inhibitory associations. It is important to clarify that this proposed model is a conceptual and mechanical framework based on the empirical correlations identified in this cross-sectional study, rather than a statistically tested causal model using structural equation modeling.

### 4.1 The burden of burden

The strong negative association between SPB and HPL (*β* = −0.38) is a critical finding. Patients with advanced cancer stages (III/IV) in our cohort reported significantly higher SPB. This aligns with findings that SPB is linked to feelings of guilt and dependency on caregivers (Cousineau et al., 2003). We extend this by proposing a hypothetical mechanism: high SPB may erode “health entitlement.” We hypothesize that patients who feel they are a financial or emotional drain on their families may sub-consciously restrict their own care needs—skipping nutritious but expensive meals, avoiding social activities that require transport, or neglecting stress management—to minimize further impact on caregivers. This potential “altruistic neglect” is a dangerous barrier to survivorship that requires family-centered interventions. Particularly in East Asian contexts characterized by collectivist values and filial piety, patients often prioritize family harmony and resource preservation over their individual needs, thereby amplifying the detrimental effects of SPB on self-care behaviors (Ko et al., 2026).

### 4.2 Resilience as a catalyst

Consistent with previous studies, resilience was positively correlated with HPL (Akbari et al., 2023; Alanazi et al., 2025). Resilient individuals in our study, particularly those with early-stage disease (Stage I/II), showed higher engagement in spiritual growth and stress management. Resilience likely facilitates cognitive reframing, allowing patients to view cancer not as a terminal sentence but as a chronic challenge requiring proactive management (Sung et al., 2021). However, our data also showed that resilience scores dropped significantly in patients with disease duration >6 months, suggesting that resilience is not infinite; it can be eroded by “chronic stress fatigue” (Alanazi et al., 2025). This highlights the need for continuous psychological “booster” sessions throughout the cancer trajectory. Importantly, our moderation analysis confirmed that robust resilience can actively buffer the severe negative impact of burden on lifestyle maintenance.

### 4.3 The stigma trap

Cancer-related stigma is prevalent and negatively correlates with lifestyle, particularly in the domains of social relations and physical activity (Williamson et al., 2022). This echoes findings by Kostorz et al. (2022), who noted that stigma contributes to social isolation. In our cohort, stigma was higher in lung cancer patients – likely linked to smoking-related stigma – compared to other cancer types (Cataldo and Brodsky, 2013; Cataldo et al., 2012; Criswell et al., 2016; Gonzalez and Jacobsen, 2012; Williamson et al., 2022; Williamson et al., 2020). Stigma creates a barrier to public exercise or joining support groups, forcing patients into a sedentary, isolated lifestyle that exacerbates physical decline (Gaddour et al., 2025). Our mediation analysis further elucidates that the harm of stigma on lifestyle is partially routed through generating a higher sense of burden.

### 4.4 Clinical implications

The results underscore that prescribing a “healthy lifestyle” (eat well, exercise more) is insufficient without addressing the psychosocial substrate. Clinicians should systematically screen for SPB using validated tools like the SPBS-CP as part of routine oncological follow-ups. Concrete interventions should include: (1) Caregiver-Patient Dyadic Therapy to openly discuss and reframe financial and emotional burden within the family unit; (2) Resilience Training focusing on self-efficacy and stress inoculation; and (3) Cognitive Behavioral Therapy (CBT) to combat internalized stigma.

### 4.5 Limitations

Several limitations must be noted. First, the cross-sectional design precludes causal inference, and the proposed mechanistic model remains conceptual rather than a statistically tested causal pathway. Second, the reliance on self-reported questionnaires introduces potential self-report bias and common method variance, which may artificially inflate the observed associations. Third, as a single-center study in Hebei, the regional specificity of the sample may limit the generalizability of the findings, particularly given the unique cultural nuances of collectivism in this area. Finally, the lack of longitudinal data restricts our ability to track how these variables dynamically evolve across different treatment phases. Future research should employ longitudinal and multi-center designs incorporating objective behavioral measures to track how these variables evolve.

## 5 Conclusion

In conclusion, this study demonstrates that self-perceived burden and cancer-related stigma are significant factors negatively associated with health-promoting lifestyles in cancer patients, while psychological resilience serves as a vital positive correlate. To improve clinical outcomes and quality of life, oncological care must integrate psychosocial interventions that specifically alleviate the patient’s sense of burden and bolster their resilience.

## Data Availability

The original contributions presented in the study are included in the article/supplementary material, further inquiries can be directed to the corresponding authors.
